# Editorial: Influential voices in soft robotics

**DOI:** 10.3389/frobt.2024.1521226

**Published:** 2024-11-08

**Authors:** Panagiotis Polygerinos

**Affiliations:** Control Systems and Robotics Laboratory (CSRL), Department of Mechanical Engineering, School of Engineering, Hellenic Mediterranean University, Heraklion, Greece

**Keywords:** editorial, influential voices, soft robotics, rising stars, future roboticists

Soft robotics, a rapidly expanding field, continues to revolutionize industries through its unique blend of compliance, adaptability, and novel applications. The “Influential Voices in Soft Robotics” Research Topic highlights emerging leaders in the domain who, despite being at the early stages of their careers, are already shaping the future of this transformative technology. This Research Topic showcases their work, encompassing advances in theory, experimental methodology, and practical applications, spanning diverse areas of soft robotics. By recognizing these promising researchers, we aim to provide a glimpse into the future of innovation in soft robotics and invite the reader to follow their careers as they show potential to define new directions for this field.

Each paper in this Research Topic provides a fresh perspective on soft robotics, highlighting new approaches to design, novel materials, and enhanced methodologies. Together, these articles not only advance the science of soft robotics but also open the door to exciting new applications. Below is a summary of the contributions from this Research Topic, each illustrating a unique facet of the field.

Addressing the theoretical foundations of soft robotics, Stella and Hughes offer a critical review of “soft robot design methodologies”. They explore the scientific motivations and challenges in designing soft robots, emphasizing the multidisciplinary nature of this endeavor. Their review covers bio-inspired design, computational methods, and emerging technologies, offering insights into how these approaches can enhance the creativity and functionality of soft robots. By formalizing the scientific questions that drive soft robot design, this paper lays the groundwork for future innovations in this field.

One of the contributions in this Research Topic is the study of “Omnidirectional soft pneumatic actuators” by Polygerinos and Moutousi. Their work presents a novel design and optimization framework using finite element methods, focusing on achieving maximum performance by considering multiple design parameters simultaneously. This approach enables users to customize soft pneumatic actuators (SPAs) for various applications, such as rehabilitation and delicate object handling. The study offers valuable guidelines for SPA design optimization, contributing to more efficient and versatile soft actuators.

Another fascinating advancement is highlighted in “self-excited pneumatic valves for soft robotics” by Nabae and Kitamura. Their research tackles the challenges of bulky driving systems in soft robotics by introducing a simplified self-excited valve system using a flat ring tube. This development promises to reduce the complexity of soft robotic systems, making them more efficient and adaptable to compact environments like pipelines or minimally invasive surgery. The success of their prototype demonstrates the potential for integrating this technology into locomotion systems, further pushing the boundaries of soft robot capabilities.

In the realm of continuum robotics, the work on “trunk-like soft robotic arms” by Tang et al. stands out. Inspired by the dexterity and strength of biological structures like elephant trunks, this study investigates how design parameters such as material hardness and actuator arrangement can enhance performance in terms of workspace, stiffness, and payload capacity. Their findings provide a comprehensive methodology for improving the design of trunk-like soft arms, offering significant potential for applications requiring high adaptability, such as search and rescue missions or hazardous environment exploration.

Finally, “visuo-dynamic self-modeling in soft robotics”, presented by Monteiro et al., pushes the boundaries of control systems in soft robotics. Their end-to-end learning-based approach models soft robotic systems dynamically using visual data, allowing for more accurate control across a wide range of tasks, from trajectory tracking to obstacle avoidance. This method is a step forward in the quest for more intelligent and autonomous soft robots, capable of adapting to complex environments in real-time.

Collectively, these contributions highlight the extraordinary potential of soft robotics while acknowledging the challenges that lie ahead. Each article in this Research Topic showcases innovative solutions to critical problems in the field, ranging from optimization techniques for soft actuators to new control methodologies and bio-inspired designs. As soft robotics continues to evolve, the researchers featured here are poised to lead the next wave of innovation, pushing the boundaries of what these adaptable systems can achieve. By amplifying their voices, this Research Topic not only recognizes their contributions but also underscores the importance of early-career researchers in shaping the future of robotics.

We invite readers to explore these groundbreaking works and follow these influential voices as they continue to redefine the capabilities of soft robotics.

